# Psoriasis therapy by Chinese medicine and modern agents

**DOI:** 10.1186/s13020-018-0174-0

**Published:** 2018-03-23

**Authors:** Shikang Meng, Zibei Lin, Yan Wang, Zhenping Wang, Ping Li, Ying Zheng

**Affiliations:** 1 State Key Laboratory of Quality Research in Chinese Medicine, Institute of Chinese Medical Science, University of Macau, Macau, China; 2 Beijing Hospital of Traditional Chinese Medicine, Affiliated with Capital Medical University, Beijing, China; 3 Department of Dermatology, School of Medicine, University of California, San Diego, La Jolla, CA USA; 4 Department of Pathophysiology, Beijing Key Laboratory of Clinic and Basic Research with Traditional Chinese Medicine on Psoriasis, Beijing Institute of Traditional Chinese Medicine, 23 Meishuguan Back Street, Dongcheng, Beijing, 100010 People’s Republic of China

**Keywords:** Psoriasis, Therapy, Chinese medicine

## Abstract

Psoriasis is a chronic, painful, disfiguring and non-contagious skin disease that has globally affected at least 200 million patients. In general, mild to moderate psoriasis patients will be treated by chemical drugs or Chinese medicine, while targeting systemic biological drugs have been successfully developed with good efficacy but high cost burden to patients with severe psoriasis. Since the underlying mechanisms of psoriasis are not well understood, in this review, psoriasis pathogenesis and clinical therapeutic principles by modern medicine and Chinese medicine are extensively described. Based on the data from the China Food and Drug Administration, the majority of chemical drugs are utilized as the topical formulations, while Chinese medicines are mainly delivered by an oral route, suggesting that the market for topical preparations of Chinese medicine to treat psoriasis is worth to exploration. Moreover, considering the unique clinical therapeutic theory and successful clinical application of Chinese medicine in the treatment of psoriasis, we believe that development of new small molecule drugs based on Chinese medicine will be a promising strategy to reduce therapeutic costs and improve safety for psoriatic patients.

## Background

### Psoriasis

Psoriasis is a chronic, painful, disfiguring and non-infectious skin disease that is believed to be an autoimmune inflammation disorder with unclear underlying mechanism [1]. Psoriasis negatively affects the quality of life of patients [2]. People of different ages and in different countries develop psoriasis. Patients with psoriasis are not often successfully surveyed because of the inconvenience of the disease, and the statistics of prevalence in different places may not be accurate. From a survey in China of 6 provinces, the overall prevalence of psoriasis was 0.47% [3]. According to current investigation, the outburst of psoriasis was not associated with gender, but mainly with the climate of the environment; that is, dry cold weather will more likely to induce psoriasis [4, 5]. Approximately 3% of people around the world have psoriasis, which is near the most common autoimmune skin disease in adults. By simple estimation, there are at least two hundred million psoriasis patients in the world [2]. Therefore, it is not just a simple health problem in a country or a region but a serious global challenge. Of note, about half of the adult patients had been reported to be sick in their childhood and they mostly fell ill around 10 years old [6, 7].

Psoriasis could be divided into five types [2]. Psoriasis vulgaris is the most common type of psoriasis. It is initially red papules or rash, then, expands gradually or merged into a flake. The surface is covered with multi-layer gray or silver white scales; Intertriginous psoriasis usually appears at the folding parts of the human body such as armpits, groin and reproductive areas, generally without any scales to be seen, the only the erythema. Guttate psoriasis usually arises in the torso among children and the onset of the disease is often related to streptococcal infection of the upper respiratory tract. Pustular psoriasis is generally seen in the palm of the hands and fingertips. The main feature of this kind is that the aggregated pustules are always filled with non-infectious pus. Erythrodermic psoriasis is the most serious type of all psoriases. It could bring about the lowering of body temperature, hypoproteinemia and high output heart failure. Generally, large areas of burn-like erythema and skin peeling are seen on the patients’ bodies.

### Modern understanding of pathologic mechanisms of psoriasis

Twenty-five years ago, people thought that, psoriasis was caused of excessive proliferation of skin cells and the chronic inflammation was just the side effect. In 1994, Krueger, a cell biologist at the Rockefeller University, started working on the origins of psoriasis. His team administered a compound that could target the immune-related cells without affecting the normal skin function of patients. The results showed a great improvement of the skin condition, and some individual cases with mild trauma were even cured. This experiment proved that psoriasis was mainly caused by immune system cells and was not the side effect of cell proliferation. The research of Krueger involved first separating the different roles of keratinocytes and T cells [8].

The main characteristics of psoriasis are excessive proliferation of keratinocytes and being easy to relapse. It is reported that this disease might involve many tissues in the muscles, joints, alimentary tract and even eyes [9]. The cardiovascular performance and abnormal metabolism phenomenon among psoriasis patients marks the important role of fat cells and vascular reconstruction related to skin inflammation, which might be mediated by sub-populations of inflammatory monocytes [9]. Nevertheless, the external factors including bacterial infection, trauma, mental pressure and genetic factors, will all lead to the onset of psoriasis [2]. Before all other symptoms are seen, at the cellular level, the accumulation of inflammatory cells headed by a large number of activated T cells and antigen presenting cells (APCs) as well as plasmacytoid dendritic cells (DCs) appear at the beginning of the disease. This is the reason why psoriasis is considered to be an autoimmune inflammatory disease [1]. According to the research progress of immunologists and psoriasis specialists in the past 20 years, the skin is essentially an active immune organ. It also becomes the carrier of several chronic inflammatory diseases like rheumatoid arthritis and systemic lupus erythematosus [10]. In recent years, studies on the relationship between autoimmune diseases and the IL-17/IL-23 axis continuously keep growing.

IL-17 is a proinflammatory cytokine produced by activated T cells, leading to the generation of inflammation [11]. IL-23 belongs to the IL-12 family and is mainly produced by activated dendritic cells, which is a heterodimer consisting of two subunits, P40 and P19. The formation pathway of Th17 cells is also called the IL-23/IL-17 inflammatory reaction axis. The general process is as follows. The initial T cells differentiate into Th17 cells with the collective effect of TGF-β and IL-6. IL-23 could improve the proliferation of Th17 cells and mediate the immune response, including secreting IL-17A, IL-17F, and IL-22. Among them, IL-22 would take part in the proinflammatory response by inducing the production of proinflammatory cytokines and chemokines [12]. Generally, activation of the IL-23/IL-17 axis could quickly gather the neutrophils and produce oxygen free radicals as well as other inflammatory mediators. As reported, autoimmune disorders were mediated by the harmful regulation of the IL-23/IL-17 axis, and this fact will create a new orientation of autoimmune disease treatment. While precisely targeting IL-23 P40, a subunit shared with IL-12, some reports showed a theoretical safety concern that it might make patients more susceptible to bacterial and other types of infections because IL-12 would induce the Th1 cells secreting IFN-γ, which is involved in anti-pathogen immune defenses [13]. Therefore, they prefer more targeting of P19 selectively. However, some other reports held their view that precisely targeting IL-23 P19 but not IL-23 P40 could lead to the blocking of the organ specific autoimmune pathologic response whose long-term immune protection cannot be guaranteed [14]. Now, the clinical results of anti-P40 treatment have already shown a good effect against psoriasis [15]. Increasing evidence has shown that the IL-23/IL-17 axis takes part in the development of psoriasis [16].

Many IL-23^+^ dendritic cells exist at the skin lesions of psoriasis. The mRNAs expression levels of subunits of IL-23 are also relatively higher [17]. The mRNA levels of Th17 type cytokines and some other kinds of chemokines were improved at the same time [18]. According to the latest articles, IFN-γ also had an strong activity inducing the hyperproliferation of keratinocytes by activating monocytes, DCs, and endothelial cells in psoriasis patients [19]. Meanwhile, IL-5 was reported to inhibit AMPs and IL-4 was reported to inhibit epidermal cornification without inducing epidermal hyperplasia in the incidence of psoriasis [20].

In summary, IL-23 secreted by dendritic cells mediate the activation of Th17 lymphocytes and the production of Th17 type cytokines, which might be the initial factor of psoriasis. At the skin lesions of psoriasis, the accumulation of cytokines, such as IL-2 and TNF-alpha, will promote the development of psoriasis. Lymphocytes in skin lesions will produce many kinds of cytokines, forming a mighty cell network headed by Th1- and Th17-type cytokines, which is also important in the pathology of psoriasis.

## Chemical and biological drugs

### Historical development

A 100 years ago, humans treated psoriasis mainly with coal tar topically. This effective therapy had been used for many years, although it is inconvenient. In 1925, a combination treatment of coal tar and UV light exposure was put into application, called “Goeckerman therapy”, which is rarely used now. From 1950 to 1970, methotrexate, corticosteroids, and psoralen with UVB treatment were put into application successively. In 1993, vitamin D3 analogs were used topically for psoriasis treatment. It had a good therapeutic effect, a high body tolerance, and the fewest side effects at that time. Acitretin was introduced in 1996 as a systemic drug and was commonly used for severe psoriasis, such as pustular and erythrodermic psoriasis. In 2003, the first systemic biologic drug in the world, Amevive, was approved by the US FDA. After 14 weeks of treatment, the Psoriasis Area Severity Index (PASI) standard ratio, which is an index combining the severity and percentage of the affected area used to express the condition of psoriasis, reached 21% [2]. Following that, many systemic biologic drugs were released, including Enbrel in 2004, Remicade in 2006, Humira in 2008 and Stelara in 2009. Among all the biologics, Stelara has even become the therapeutic standard for judging new psoriasis systemic biologic drugs, because after 28 weeks of treatment, the PASI 75 standard ratio, which is the percentage of patients achieving a reduction of at least 75% in their baseline PASI at a given time, could reach approximately 80% [21, 22].

### Current treatment

Currently, there are many types of treatments for psoriasis of different degrees, as shown in Table 1. Mild to moderate psoriasis patients will usually use topical drugs, regulating gene transcription, to inhibit the proliferation of cells and boost the differentiation of keratinocytes. In terms of severe psoriasis patients, physical treatment combined with oral systemic drugs that regulate epidermal cell differentiation and proliferation such as Methotrexate, Acitretin or Cyclosporine were applied [23]. Ultraviolet radiation b (UVB) exposure at a wavelength of 311 nm has now become the major physical therapy for the treatment of psoriasis. Its remission period is short, but long-term application causes skin aging, pigmentation, skin cancer and increasing the risk of cataracts. When treating severe psoriasis vulgaris by irradiating 3–4 times a week, and the efficacy could reach 80% [24].

**Table 1:** Common medication of psoriasis

Psoriasis degree	Suitable formulation	Common drugs	Drug action	Formulation features
Mild Moderate	Topical drugs	Corticosteroids Retinoids Calcineurin inhibitors Vitamin D analogues	Regulate genes transcription Inhibit the proliferation of cells Boost the differentiation of keratinocytes, etc.	Avoidance of first pass metabolism Avoidance of extreme condition in GI Reduction of systemic toxicity Direct access to target of skin High patient compliance Limitation in molecular weight/Log P of drugs
Severe	Systemic chemical drugs (oral)	Methotrexate Acitretin Cyclosporine	Immunosuppressive effect Anti-inflammatory Inhibit PDE-4, etc.	Convenient, Safe Slow absorbing velocity Unfit to unconscious patients
	Biologics (injection)	Ustekinumab Secukinumab Etanercept Stelara	Target IL-17, IL-23, TNF-α	High targeting specificity Few adverse reactions Safe Extremely expensive

Biologics that work against IL-17, IL-23, or TNF-α are usually injected as systemic drugs because of their good efficacy and safety. The efficacy is outstanding and fits nearly all conditions of psoriasis. Biologics work quickly, have a long duration, have a long treatment cycle and do not produce relapse. There are still a few limitations about patient applicability. Generally, the age should be between 18 and 65 years; psoriasis should be diagnosed for more than 6 months; the psoriasis affected area should be at least 10% and the PASI score should be over 12 during the baseline period. There should be no potential or active tuberculosis before screening. However, the biggest reason hindering the promotion of biologics is their high cost. Although only administered once every 8 weeks, the cost of the therapy of each time reached ~ 2100 dollars. Therefore, only patients with severe psoriasis are recommended for biologics.

Over all, the final purpose of any kind of psoriasis treatment was to eliminate all the symptoms on the skin. The minimum requirement for therapy evaluation was set to be over 50% reduction of the PASI baseline [25]. If this goal could not be reached, then the therapy should be modified by the way of adjusting the dosage, turning to another treatment or using a combination therapy. In terms of moderate to severe psoriasis, phototherapy combined with systemic oral drugs or biologics is usually recommended. Griffths et al. [26] reported that a combination of retinoid and PUVA was more effective than each given alone, and this combination had the potential for lowering the cumulative UVA dosage. They also reported that phototherapy combined with a vitamin D3 analog such as calcipotriol was more effective than either alone. Roman et al. [27] reported a successful case with a complete clearance using a combination of acitretin and apremilast on patients with severe palmoplantar psoriasis. AbuHilal et al. [28] reported several cases using apremilast combined with other therapy, including NB-UVB, methotrexate and acitretin, of which 81% achieved PASI 75 at week 12 after the combination was applied.

### Common topical chemical drugs

According to the statistical induction of CFDA about domestic psoriasis chemical drugs, as shown in Table 2, among all 890 drug products recorded in the market, only 169 of them are systemic drugs, while most of them are topical formulations, including creams, ointments and solutions [29]. The most common topical chemical drugs are listed and described below.

**Table 2:** Chemical drugs for topical delivery to treat psoriasis

Drug	Formulas approved by FDA	Dosage	Indications	Adverse reactions and precautions
Beclometasone Dipropionate^a^	Cream (41)^e^, ointment (1), spray (8), plasters (2)	2–4 times/day, less than 45 mg/week	Contact eczema, atopic dermatitis, neurodermatitis, pruritus, psoriasis, sclerosing atrophic moss, lichen planus, discoid lupus erythematosus, seborrheic dermatitis, hypertrophic scars	Short-term application: burning, tingling, itching Long-term application: skin telangiectasia, skin atrophy, atrophy pattern, secondary purpura, ecchymosis, skin fragility, hirsutism, folliculitis, milia, skin bleaching, systemic adverse reactions (absorbed too much)
Fluocinonide^a^	Cream (72), ointment (1), tincture (27), liniments (1)	2–3 times/day, less than 12.5 mg/week		
Halcinonide^a^	Cream (27), ointment (10), solution (37)	Twice/day		
Hydrocortisone Butyrate^a^	Cream (11)	Twice/day		
Triamcinolone Acetonide^a^	Spray (5), cream (86), paste (2), ointment (22)	2–3 times/day		
Methoxsalen^b^	Solution (12)	Once/day, apply UVA 2–4 h later	Psoriasis vulgaris and acne vulgaris	Erythema, skin pigmentation, itching, redness, blisters, pain, scaling
Tazarotene^b^	Cream (2), gel (3)	Apply half an hour before sleep, then wash off.		Itching, erythema, burning sensation, skin irritation, dryness and edema
Tretinoin^b^	Cream (35)	1–3 times/day		Burning, erythema and desquamation, but gradually disappear
Tacrolimus^b^	Ointment (4)	Twice/day	Patients unfit traditional therapies due to potential risk	Rosacea, edema at the site of administration
Calcipotriol^c^	Ointment (2)	1–2 times/day, less than 100 g/week	Psoriasis vulgaris	Pruritus, burning sensation, tingling, dry skin, erythema, contact dermatitis, eczema
Biphenyl benzazole^d^	Gel (5), solution (8), spray (1), cream (39), film (1)	Once/day	Hand skin fungi, yeasts, molds, fungal skin diseases caused by pityrosporum and infections caused by Corynebacterium parvum	Pain and peripheral edema, contact dermatitis, erythema, pruritus, rash, urticaria, blisters, peeling, eczema, dry skin, skin irritation, skin impregnation, burning sensation
Clotrimazole^d^	Solution (32), cream (102), liniments (7), film (3)	2–3 times/day	Tinea corporis, tinea corporis, tinea capitis, tinea pedis, tinea versicolor, tinea capitis, and candidiasis parvitis and candidal vulvovaginitis	Itching, tingling, erythema, edema
Econazole Nitrate^d^	Solution (9), spray (4), cream (42)	Twice/day		Itching, tingling, erythema, edema
Terbinafine Hydrochloride^d^	Cream (37), liniments (2), spray (7), gel (11)	Once/day		Burning sensation, rash, pruritus

^a^Corticosteroids

^b^Small molecule inhibitors

^c^V_D_ analogues

^d^Broad spectrum antimicrobials

^e^Cream (41) means 41 cream formulations had been proved by CFDA

Corticosteroids, such as Fluocinonide, are used for the treatment of itching and noninvasive skin diseases. These anti-inflammatory anti-allergy drugs could inhibit the proliferation of connective tissue, reduce the permeability of capillaries and cell membranes, reduce inflammatory exudation, and inhibit the formation and release of histamine and other inflammatory mediators.

Retinoids, such as tazarotene, regulate the abnormal differentiation of keratinocytes, alleviate proliferation and speed up the inflammation subsiding. The specific performance after treatment include down-regulation of cell differentiation markers to reduce over-differentiation, inhibition of cell proliferation markers, reduction of the expression of inflammatory markers and up-regulation of tazarotene induced genes to against the proliferation.

Calcineurin inhibitors, such as tacrolimus, belong to macrolide antibiotics. It is a kind of new powerful immunosuppressive agent that are fit for patients who cannot receive traditional therapy and those severe patients who cannot tolerate the treatment program. On a molecular level, tacrolimus is combined with the cellular protein, FKBP12. The FKBP12-tacrolimus complex will specifically combine and inhibit calcineurin, which will inhibit the calcium ion-dependent message conduction pathway in cells, preventing the transcription of the discontinuous lymphokine gene.

Vitamin D analogs, such as calcipotriol, have a strong affinity to the calcitriol receptor. They can decrease the content and distribution of IL-6 as well as the amount of T lymphocytes in the activated epidermis in psoriasis patients. The mechanisms for inhibiting the hyperplasia and inducing the differentiation of skin cells are still not clear and are possibly influenced through the JAT/STAT signaling pathway.

## Psoriasis therapy based on “treatment from blood aspect”

Different from previous understanding of psoriasis, in ancient books of traditional Chinese medicine (TCM), for the psoriasis-like diseases, pathogenesis was thought to be the exogenous evils invading and battling in human blood. As recorded in the ancient book *Zhu Bing Yuan Hou Lun* “Blood dryness symptom, caused by all evils, pathogenic wind and dampness attacking and stalling between skin and fascia, together with evil cold, defeated positive Qi of the blood” [30]. Pathogenic wind dries the fluid in the blood, by which skin and muscle could not be nourished and moisturized. As the same sayings in *The essence of medicine is the secret of the heart of surgery* [31]:“Psoriasis, either skin suffered from pathogenic wind, or lost nourishment from blood dryness”.

After 1949, a number of experts, such as Dr. Bing-nan Zhao, Ren-kang Zhu and Qi-fengJin, advocated “treatment of psoriasis from the blood aspect”. For example, Zhao [32, 33] proposed the evil fire should be caused by internal heat from emotional stress, which blocked Qi movement, and excessive Qi gathered together to produce fire. As a result, the evil fire entered and stalled in blood circulation; or by stagnated heat derived from Qi dysfunction between the spleen and stomach, due to an improper diet, which caused disease when suffering from exogenous evil fire once again. During a long disease course, *Yin* and blood were exhausted, which led to pathogenic wind blowing or blood circulation stagnation. At last Qi and blood coagulated and blocked the meridians, which resulted in skin and muscle loss of nourishment.

### The enrichment and development of psoriasis vulgaris based on “treatment from blood aspect”

“Treatment from blood aspect” is the principal diagnosis system of psoriasis vulgaris, which can be used as an overall differentiation system together with other differentiation methods such as six climatic evils, visceral syndrome, and/or poisonous pathogenic. For example, Zhu [34] first proposed that the treatment of psoriasis should be based on the principle “Treatment from blood aspect, combined with other ways”, and advocated that the differentiation of psoriasis should combine with other syndrome differentiation systems, such as expelling wind and detoxification. Jin [35] stated the pathogenesis of psoriasis should be mainly divided into four aspects, including invasion of exogenous pathogenic factors, entrance of heat into the blood phase, obstruction of heat-toxins on the collaterals and *Yin* deficiency and blood dryness. Therefore, the syndrome falls into four categories: blood heat, dampness heat, blood dryness and blood stasis. Zhang [36] suggested that except for blood heat, blood dryness and blood stasis, there were still dampness heat and heat-toxin syndromes. The former one usually occurred in exudation type of psoriasis, and latter one was caused by acute tonsillitis or upper respiratory tract infection. Xu [37] reported the aeropathic factors were the invasion of pathogenic wind, cold, dampness, heat and dryness, and the intrinsic factors were congenital blood heat, improper diet and emotional hurt. In the early stages, blood symptoms such as blood heat, blood dryness and blood stasis were the main clinical manifestations. However, after a long disease course, the function of Zang and Fu declined, among them, and the liver and kidney showed the most prominent damage. Ma [38] thought psoriasis was caused by blood stasis and heat-toxin. Cooling-Blood and detoxification should be used in the early onset, and after a long duration, promoting-blood circulation and detoxification was available. In short, all the methods are beneficial supplements to the “treatment from blood aspect”.

In addition, understanding the pathogenesis of blood aspect got improved and was supplanted with considering different physical states. If the disease exacerbated in winter, complicated with lassitude, lack of *qi* and no desire to speak, spontaneous perspiration, drowsiness, thin and weak pulse, cold limbs, low back cold, tastelessness and lusterless complexion, it should be treated by warming *yang* based on “treatment from blood aspect” to improve the curative effect. If chronic plaque psoriasis with skin lesions, less sweat and feeling oppressed was accompanied by physical obesity, dizziness, drowsiness, heavy body fat, elevated blood lipids and other characteristics, except the existence of blood stasis symptoms, the patients had phlegm blocking and disharmony between *Ying* and *Wei*, with phlegm and blood stasis. Here, “Warming *Yang* and harmony *Ying*, cooling blood and improving blood circulation” therapy should be applied under the guidance of cold and hot medicine equivalence.

### Research progress on the syndrome evolution of psoriasis vulgaris based on “treatment from blood aspect”

The “treatment from blood aspect” of psoriasis vulgaris should pay attention to the conversion and evolution of syndromes. First, Deng [39] reported that there are three basic syndromes, including blood heat, blood dryness and blood stasis, by large-scale epidemiological survey. Second, the distribution of the three syndromes was closely related to the stage of the disease. Blood heat syndromes were commonly seen in a progressive stage, blood dryness in the extinction stage and blood stasis in a stationary stage. However, some of patients showed three instability syndromes, such as blood heat with blood dryness, blood dryness with blood stasis, as well as blood stasis with blood heat. Among them, the conversion of blood heat with blood dryness syndrome was the most common transformation type.

Li et al. [40] employed a cross-sectional survey of 500 cases of psoriasis vulgaris in the Central China region. The results showed that the main syndromes of patients are blood stasis, blood dryness, blood heat, blood heat with blood stasis, blood heat with blood dryness and blood stasis with blood dryness. In all cases, patients with six types of blood syndrome in stable and unstable stages accounted for 91%. Among them, the blood stasis syndrome accounted for 57%. In the progression stage, TCM syndromes were blood heat and blood heat with blood stasis, while in stationary stages, it was blood stasis with blood dryness, then in extinction stage it was blood dryness. Meanwhile, the longer course it took, the more blood stasis and dryness syndromes appeared.

Zhang et al. [41] employed a cross-sectional survey on 2651 cases of psoriasis vulgaris in three hospitals of Traditional Chinese medicine in the Beijing area. The data demonstrated that the blood heat syndrome is the most common in psoriasis vulgaris, followed by blood dryness and blood stasis. The distribution of syndromes is closely related to the stage of the disease. The syndromes of blood heat, blood dryness and blood stasis are commonly seen in progression, catagen and the stationary stage, respectively. The PASI score, severity index of the disease is closely related to the syndrome distribution. With aggravation of the disease, the PASI value increased, the proportion of both blood heat and blood stasis syndrome also increased, while the proportion of blood dryness syndrome decreased.

## Treatment of psoriasis with traditional Chinese medicines

The syndrome differentiation and treatment of psoriasis mainly involve removing pathogenic heat from the blood and toxic material from the body, promoting blood circulation and removing blood stasis, nourishing blood and removing toxins, where the central idea of treatment is “treatment from blood aspect”. Therefore, Chinese medicines for regulating blood conditions are considered to be effective, including Chinese medicines that can cool blood, nourish blood and promote blood circulation. Cooling-blood Chinese medicines are divided into those that can cool blood to be antipyretic and those that can cool blood to stop bleeding. Among all the blood-regulating Chinese medicines, those most widely used for treating psoriasis in practice are *Arnebiae Radix* (Zicao), *Paeoniae Radix Alba* (Baishao), *Paeoniae Radix Rubra* (Chishao), *Spatholobi Caulis* (Jixueteng), *Moutan Cortex* (Danpi), *Curcumae Rhizoma* (E’zhu) and *Salviae Miltiorrhizae Radix and Rhizoma* (Danshen). In addition, since psoriasis has the characteristics of toxin-damage of the choroid, antipyretic-detoxicate Chinese medicines are used frequently [42].

### Current clinical practice of anti-psoriatic traditional Chinese medicines

Various remedies used to treat psoriasis were recorded in Chinese medicinal classics, like *TaiPingShengHui Formulas* and *PuJi Formulas*, which can be divided into two parts as formulas for topical or for oral use, respectively. Topical remedies involve preparations manufactured into traditional dosage forms, like creams, oils, unguentum, plaster and lotion decoctions, while oral drugs involve those prepared into decoctions, tablets and pulvis, etc. The use of traditional herbal compound prescriptions for psoriasis treatment is under the guideline of “treatment from blood aspect”, Therefore, herbs that are targeted to “blood” and detoxification are usually used. A clinical study on 675 cases [43] made a comparison between *Yinxieling Ointment*, a topical prescription [Mustard gas, R*adix Sangusorbae* (Diyu), *Radix Scutellariae* (Huangqin), *Cortex Phellodendri* (Huangbai)] and Dichlorodiethyl sulfide, and they found comparatively equal efficacy. For oral prescriptions, the clinical efficacy of *HuoXueSanYu Decoction* [44] [*Rhizoma Sparganii* (Sanleng), *Curcumae Rhizoma* (E’zhu), *Semen Persicae* (Taoren), *Flos Carthami* (Honghua), *Spatholobi Caulis* (Jixueteng), *Ramulus Euonymi* (Guijianyu), *Herba Hedyotis* (Baihuasheshecao), *Salviae Miltiorrhizae Radix and Rhizoma* (Danshen), and *Pericarpium Citri Reticulatae* (Chenpi)] were found to be comparable to acitretin (10 mg, b.i.d.), for the treatment of psoriasis vulgaris. *JiaWeiHuangLianJieDu Decoction* [45] [*Radix Scutellariae* (Huangqin), *Cortex Phellodendri* (Huangbai), *RhizomaCoptidis* (Huanglian), etc.] was reported to alleviate the symptoms in 45 of 50 psoriatic subjects.

In most cases, the formula for anti-psoriatic treatment consists of different kinds of herbs, which are used as adjuvant therapy or joint with other drug as combined treatment. Anti-psoriatic herb compound prescriptions commonly combine with Narrow Bound Ultra Violet B(NB-UVB) light to achieve higher efficacy. A study [46] analyzed the effect of *Tuiyin Decoction* combined with NB-UVB, where one group used combined therapy and two control groups applied NB-UVB or *Tuiyin Decoction*, respectively, and efficiency rates were revealed to 95.3, 67.4 and 72.1%, respectively, indicating that joint treatment has much higher efficacy. Some psoriasis cases are also treated by chemical drugs jointly with Chinese herbal formulas, such as corticosteroids, calcineurin inhibitors and so on. A study [47] demonstrated that calcipotriol betamethasone ointment combined with *PSORI*-*CM01 Decoction* [*Paeoniae Radix Rubra* (Chishao), *Rhizoma Curcumae* (Erzhu), *Sarcandra* (Caoshanhu), *Radix Glycyrrhiza* (Gancao), *Fructus Mume* (Wumei), *Arnebiae Radix* (Zicao), and *Rhizoma Smilacis Glabrae* (Tufuling)] performed better efficacy and a lower relapse rate than combined with an oral placebo. *ZaoShiKuShen Decoction* [48] [*Radix Sophorae Flavescentis* (Kushen), *Coix chinensis Tod.* (Yiyiren), *Rhizoma Smilacis Glabrae* (Tufuling), *Cortex Phellodendri* (Huangbai), etc.] was jointly applied with 0.03% Tacrolimus, which showed 14% higher efficacy than that of 0.03% tacrolimus alone in a 100-patient clinical trial.

Some of the traditional prescriptions or active ingredients extracted from Chinese herbs have been developed into hospital agents with good efficacy. For instance, an *Indigo naturalis* composite ointment [consisting of *Indigo naturalis* (Qingdai), *Scutellaria baicalensis Georgi* (Huangqin) and *Cortex phellodendri* (Huangbai)] exhibited a successful treatment on pediatric psoriasis [49]. New Pulian Ointment consisting of Huangqin and Huangbai has successfully been used to treat psoriasis of blood-heat syndrome [50]. Herose capsules consisting of (*Rhizoma Zingiberis* (Ganjiang), *Radix Salviae Miltiorrhizae* (Danshen), *Radix Astragali* (Huangqi), *Ramulus Cinnamomi* (Guizhi), *Radix Paeconiae Alba* (Baishao), *Radix Codonopsis Pilosula* (Dangshen) and *Semen Coicis* (Yiyiren)) were reported to have better efficacy on plaque psoriasis [51].

In addition, drugs approved by CFDA have also been studied, which contained herbal prescriptions and active composites extracted from herbs in various dosage forms, such as tablets, pills, creams, ointments, patches and injections. In the 34 approved drugs, the majority of them were orally administered two or three times per day, while 17 of them were the same extraction prepared in different dosage forms, such as formula Fufangqingdai, which has been prepared into tablets (Approved Number: Z20150034), pills (Approved Number: Z61020964), condensed pills (Approved Number: Z20080269) and capsules (Approved Number: Z20010157). There were also two injections, one containing *Radix Sophorae Flavescentis* (Kushen) (Approved Number: Z14021231), another involving *Fructus Psoraleae* (Buguzhi) (Approved Number: Z41022361) and six topical agents. However, from 2004 to June 2012, the National Adverse Drug Reaction Monitoring Center has revealed 344 cases that appeared to have adverse reactions in that digestive system, skin and its affiliated glands, and the nervous system after being treated with systemic administered prescriptions, and 23 severe cases involved medicinal liver damage and gastrointestinal bleeding. Compared to systemic preparations, those for topical use showed less adverse events including hotness, redness, dryness or itching on the administered skin, which are usually reversible or self-healing.

### Modern studies on anti-psoriatic extraction from Chinese medicines

With huge potential for treating psoriasis, novel drugs involving pure ingredients extracted from Chinese herbs also have been explored. Curcumin, which is an active constituent in *Curcuma longa* L., has been developed into various kinds of formulations due to its inhibition of several inflammatory enzymes. Studies have found that it exerted anti-psoriatic action probably via suppressing the expression of VEGF and iNOS [52]. Additionally, curcumin was reported to show anti-inflammatory and growth suppressive effects in TNF-α treated HaCaT cells through inhibition of the NF-κB and MAPK pathways [53]. Curcuminoid C3 complex capsules are an oral drug candidate, which is under a phase II clinical trial to assess its safety and efficacy [54]. Fufangezhu Oil Cream [55] and ethanolic extract of ‘Ezhu’ [56] were both applied in animal tests and showed better efficacy. Topical nano-emulsion of turmeric oil was employed in both in vivo and in vitro animal experiments and was found to have high potential to treat psoriasis [57]. Our previous work has applied curcumin-load PLGA nanoparticles hydrogel topically to treat psoriatic mice with good bioactivity [58].

Moreover, we have performed extensive studies to understand the underlying mechanisms of single constituents isolated from effective Chinese medicines on psoriasis treatment. For example, our recent studies revealed that indirubin, the active component of *Indigo naturalis* (Qingdai), significantly mitigated psoriatic lesions induced by imiquimod in mice. Meanwhile, it could suppress infiltration of inflammatory cells, proliferation of epidermal cells and expression of pre-inflammatory factors. In addition, in vitro studies demonstrated the inhibition effect on the expression of IL-17A and phosphorylation of STAT3 and JAK3, indicating that the expression of IL-17A from γδ T cells and immuno-inflammatory reactions can be inhibited by indirubin probably via mediating the JAK3/STAT3 signaling pathway [59]. *Paeonia lactiflora Pall.* (Shaoyao), whose active compound is paeoniflorin, can significantly alleviate imiquimod-induced psoriatic lesions and inhibit the infiltration of inflammatory cells as well as the proliferation and differentiation of epidermal cells. Furthermore, it mainly targeted the regulation of Th cell factor expression, and inhibited the excretion of relevant cytokines probably via modulating the phosphorylation of STAT3 [60]. Astilbin, the active composite extracted from *rhizoma Smilacis Glabrae* (Tufuling), showed suppressive effects on the differentiation of Th 17 cells and γδ T cells. Therefore, the content of IL-17 in lesions and peripheral blood was mediated. However, the specific underlying molecular mechanics remains unclear [61]. *Tripterygium wilfordii Hook.* (Leigongteng) is a traditional medicinal herb that has been used for several centuries in China. Multi-glycoside from this herb could effectively disturb the formation of skin lesions in imiquimod-induced mice and inhibit the proliferation of epithelial cells and infiltration of T cells in lesion sites via downmodulating Th 17 function [62]. *Arnebiae Radix* (Zicao), a blood-regulating herb, and its ingredient β, β-dimethyl acryloyl alkannin has demonstrated suppressing psoriasis-activated dendritic cells and inhibiting mRNA expression of inflammatory factors due to curbing the TLR7/8 pathway [63, 64].

## Conclusions and perspectives

Psoriasis not only affects people with itching and pain but also causes serious distress in their daily lives. Because of the abnormal appearance and texture of the skin, it is likely that psoriasis patients will be less likely to take part in social activities because of depression, so apparent skin treatment is more important. According to the CFDA data, the chemical formulations of psoriasis treatments are mainly topical drugs, while the traditional Chinese medicine formulations are mainly oral systemic drugs, suggesting that the market of topical preparations of Chinese medicine for the treatment of psoriasis is still waiting for exploration. At present, the most effective drug is biologics, but their cost, relative to chemical drugs or traditional Chinese medicine, is too high to be used for long term treatment. Looking for new technologies to reduce production costs is definitely a development trend for monoclonal antibody drugs. On the other hand, compared to chemical and biological treatments, traditional Chinese medicine and their active extracts are much cheaper with fewer adverse reactions, which is more suitable for the treatment of less severe psoriasis. Therefore, the topical use of Chinese medicine or their small-molecule extractions for the treatment of psoriasis is a potentially promising new area for future exploration.
